# Impact of inpatient volume on residents’ In-training examination scores and burnout in Japanese community hospitals: a nationwide cross-sectional study

**DOI:** 10.1186/s12909-026-08664-3

**Published:** 2026-01-24

**Authors:** Kosuke Ishizuka, Yuji Nishizaki, Koshi Kataoka, Taro Shimizu, Masanori Nojima, Yu Yamamoto, Kiyoshi Shikino, Sho Fukui, Kazuya Nagasaki, Hiroyuki Kobayashi, Mitsuyasu Ohta, Yasuharu Tokuda

**Affiliations:** 1 Department of General Medicine, Yokohama City University School of Medicine, 3-9 Fukuura, Kanazawa-ku, Kanagawa Yokohama, Japan; 2 Department of General Medicine, Yokohama City University Medical Center, Yokohama, Kanagawa Japan; 3 Division of Medical Education, Juntendo University School of Medicine, Tokyo, Japan; 4 Department of Diagnostic and Generalist Medicine, Dokkyo Medical University Hospital, Tochigi, Japan; 5 Center for Translational Research, The Institute of Medical Science Hospital, The University of Tokyo, Tokyo, Japan; 6 Division of General Medicine, Center for Community Medicine, Jichi Medical University, Tochigi, Japan; 7 Department of General Medicine, Chiba University Hospital, Chiba, Japan; 8 Department of Community-oriented Medical Education, Graduate School of Medicine, Chiba University, Chiba, Japan; 9 Health Professional Development Center, Chiba University Hospital, Chiba, Japan; 10 Department of Emergency and General Medicine, Kyorin University Hospital, Tokyo, Japan; 11 Department of Internal Medicine, Mito Kyodo General Hospital, University of Tsukuba, Ibaraki, Japan; 12 Muribushi Okinawa Center for Teaching Hospitals, Okinawa, Japan; 13 Tokyo Foundation for Policy Research, Tokyo, Japan

**Keywords:** Burnout, Clinical training, GM-ITE, Inpatient volume

## Abstract

**Background:**

The optimal hospital size between adequate clinical training and resident well-being remains underexplored, particularly within Japan’s unique medical training environment. This study examined the relationship between residents’ nationwide General Medicine In-Training Examination (GM-ITE^®^) scores and their mental health to evaluate the validity of inpatient volume as a criterion for training hospital designation.

**Method:**

We conducted a nationwide cross-sectional study of 7,498 postgraduate year (PGY)-1 and PGY-2 residents from 608 community-based hospitals in Japan who participated in the GM-ITE^®^ from January 17 to 30, 2024. The GM-ITE^®^ is a highly reliable test for evaluating residents’ basic clinical skills. The 2023 GM-ITE^®^ included 80 multiple-choice questions with a maximum score of 80. In the Mini Z 2.0 survey, a score of 3 or more was defined as the presence of burnout symptoms. Training facilities were categorized into four groups by yearly inpatient volume: Very Low (< 3,000), Low (3,000–5,999), Moderate (6,000–9,999), and High (≥ 10,000). We explored the relationships using multivariable analysis between GM-ITE^®^ score, burnout symptoms, and training environment.

**Result:**

The GM-ITE^®^ scores in the Very Low-Volume group were significantly higher than those of the Low-Volume group (45.8 ± 7.3 vs. 44.3 ± 7.1; adjusted coefficient (AC): 2.007; *p* = 0.021), but did not differ significantly from those of the Moderate- (45.8 ± 7.3 vs. 44.9 ± 6.7; AC: 1.637; *p* = 0.060) or High-Volume (45.8 ± 7.3 vs. 46.2 ± 6.9; AC: 1.638; *p* = 0.082) groups. The prevalence of burnout symptoms among residents in the Very Low-Volume group, did not differ significantly from that of residents in the Low- (13.5% vs. 13.1%; adjusted odds ratio (aOR): 0.974; *p* = 0.940), Moderate- (13.5% vs. 12.8%; aOR: 0.967; *p* = 0.923), and High-Volume groups (13.5% vs. 12.1%; aOR: 0.994; *p* = 0.986).

**Conclusion:**

Residents at Very Low-Volume hospitals achieve comparable GM-ITE^®^ scores and mental health outcomes to those of residents at larger hospitals. Even Very Low-Volume hospitals are individually assessed, and if recognized as having an educational environment where residents can acquire basic clinical skills, it may be appropriate to certify them as core clinical training hospitals. The educational environment in Very Low-Volume hospitals could be enhanced by supporting residents’ mental health and advancing international clinical training programs.

**Supplementary Information:**

The online version contains supplementary material available at 10.1186/s12909-026-08664-3.

## Background

Japan has 82 medical schools offering a six-year curriculum which begins immediately after completing high school [1, 2]. Many students participate in a two-year clinical training program after completing medical school [1, 2]. This post-graduate clinical training, made mandatory in April 2004, comprises a 96-week comprehensive rotation training. Currently, residents rotate through internal medicine (at least 24 weeks), emergency medicine (at least 12 weeks), surgery, pediatrics, obstetrics and gynecology, psychiatry, and community medicine (at least 4 weeks each), with the remaining time allocated to elective subjects, providing residents with diverse clinical experience [3, 4]. The Ministry of Health, Labor and Welfare (MHLW) has established clinical training guidelines and oversees training programs to ensure that residents acquire basic clinical knowledge, skills, communication abilities, professionalism, and ethical awareness [5]. A computerized nationwide matching system allows medical students to apply to any clinical training hospital in Japan [5]. In 2023, 1,029 hospitals provided clinical training. On completing the two-year clinical training program, many residents begin specialized senior resident training. A minimum inpatient volume is essential to provide residents with diverse clinical experience enabling them to acquire basic clinical knowledge and skills. Training in hospitals with a high inpatient volume offers residents opportunities to enhance their clinical skills through exposure to a wide range of cases. Additionally, high-volume environments help residents acquire a broader spectrum of clinical management skills [6]. However, a high inpatient volume can increase residents’ workload, increasing the risk of burnout and potentially lowering the quality of education [7, 8]. Therefore, evaluating the impact of inpatient volume on the effectiveness of resident education and their well-being is crucial for improving the quality of clinical training programs [9]. The Japanese clinical training system has specific criteria for designating clinical training hospitals. The Ministerial Ordinance on Clinical Training, specified in Article 16 − 2, Paragraph 1 of the Medical Practitioners Act, requires that core clinical training hospitals have a minimum of 3,000 inpatients annually [9]. Currently, facilities with a yearly inpatient volume of under 3,000 can be certified if they provide an educational environment where basic clinical skills can be acquired based on an individual assessment [9]. To our knowledge, the threshold of 3,000 inpatients annually is not based on objective data. Due to changes in Japan’s population dynamics and social structures, there is a redistribution of hospital beds for acute, sub-acute, convalescent, and long-term care, and a review of the world’s largest number of hospital beds from a medical economic perspective, and clinical training hospitals are also being forced to change. Therefore, evaluating the suitability of hospitals with fewer than 3,000 yearly inpatient volumes as core clinical training hospitals remains imperative. In this context, we deliberately used the current regulatory cut-off of 3,000 yearly inpatient volumes to define Very Low-Volume hospitals so that the empirical validity of this criterion could be evaluated in relation to residents’ GM-ITE^®^ scores and burnout symptoms [9]. 

The quality of clinical training is also dependent on the training guidance system, clinical training support system, and availability of academic journals [10–12]. A well-structured guidance system enables residents to enhance their clinical skills and decision-making abilities through feedback from senior physicians and supervisors [10]. Conversely, the quality of clinical training may deteriorate if supervision and feedback are inadequate. Providing psychological and physical support is crucial to providing a supportive learning environment. If training schedules are appropriately managed and mental health support systems are established, residents can maintain their well-being and learn effectively [11]. Moreover, access to resources such as academic journals enhances the quality of clinical training. Online access to academic journals and educational materials is essential for self-directed learning [12]. These enable residents to rapidly gather evidence to address challenges encountered in daily practice and enhance their clinical decision-making capacity [12]. However, the relationship between inpatient volume and the training environment for residents, including the training guidance system, clinical training support system, and availability of academic journals, remains underexplored.

This study aimed to provide a comprehensive analysis of the characteristics of community-based clinical training hospitals in Japan according to their yearly inpatient volume, to evaluate the relationship between the nationwide General Medicine In-Training Examination (GM-ITE^®^) scores and the mental health of residents, and to assess the validity of yearly inpatient volume as a criterion for designating clinical training hospitals. It also investigated the relationship between the educational environment in clinical training hospitals and inpatient volume, and their impact on the acquisition of basic clinical skills and mental health, to provide empirical evidence for improving the training environment for residents. The study was intended to provide foundational data for policy decisions to enhance the quality of clinical training and mental health support, including burnout prevention.

## Methods

### Study design

We conducted a nationwide cross-sectional study of the characteristics of clinical training hospitals. We analyzed data from residents who participated in the General Medicine In-Training Examination (GM-ITE^®^) at the end of 2023, and explored the association between yearly inpatient volume at clinical training hospitals and residents’ GM-ITE^®^ scores and mental health. This study adhered to the Strengthening the Reporting of Observational Studies in Epidemiology (STROBE) guidelines for cross-sectional studies [13]. 

### Context and participants

Residents participating in the GM-ITE^®^ were affiliated with either university or community-based hospitals. This survey targeted residents affiliated with community-based hospitals. As university hospitals tend to have a high yearly inpatient volume, residents affiliated with university hospitals were excluded. A total of 7,498 residents working at 608 community-based hospitals in Japan participated in the GM-ITE^®^ from January 17 to 30, 2024. Those who did not consent to participate or for whom complete data on the clinical training environment were unavailable were excluded.

### GM-ITE^®^

In the United States, an in-training examination, the Residency Internal Medicine In-Training Examination (IM-ITE), is conducted to assess clinical knowledge during training [14–16]. In 2011, the GM-ITE^®^, modeled on the IM-ITE, was developed and introduced in Japan. It has subsequently become a nationwide examination in Japan, with over half of all residents participating. The GM-ITE^®^ is administered at the end of postgraduate year (PGY)-1 and PGY-2 and consists of questions aligned with the MHLW’s Clinical Training Guidelines for Physicians [17, 18]. It provides an accurate assessment of basic clinical skills [17, 18]. It is organized into four categories: “Medical Interview and Professionalism,” “Symptomatology and Clinical Reasoning,” “Physical Examination and Clinical Skills,” and “Disease-specific Topics,” and also includes subjects such as internal medicine, surgery, obstetrics and gynecology, pediatrics, psychiatry, emergency medicine, and community medicine, related to the clinical rotation. GM-ITE^®^ is not merely a test of medical knowledge but also evaluates the practical clinical skills and management abilities. It is a computer-based test (CBT), leveraging CBT strengths such as video-based questions to assess residents’ clinical skills. The GM-ITE^®^ aims to facilitate improvements in clinical training programs by providing an objective and reliable evaluation of residents’ basic clinical skills to both residents and training program managers [19]. For residents, the GM-ITE^®^ serves not as a pass-fail examination but rather as a means to identify proficiency in basic clinical skills and weaknesses in specific clinical fields [20]. The 2023 GM-ITE^®^ comprised 80 multiple-choice questions, categorized as follows: “Medical Interview and Professionalism” (8 questions), “Symptomatology and Clinical Reasoning” (18 questions), “Physical Examination and Clinical Skills” (18 questions), and “Disease-specific Topics” (36 questions). Each question was worth one point, resulting in a maximum score of 80. Although the primary language of the GM-ITE^®^ was Japanese, 10 out of the 80 questions were presented in English, highlighting the importance of bilingual proficiency in medical practice.

### Data collection

We analyzed facility data, including the number of permitted beds, yearly inpatient volume, annual number of ambulances, annual number of outpatients, average length of hospital stays (for general beds), number of doctors, number of nurses, number of computerized tomography (CT) and magnetic resonance imaging (MRI) scans, in addition to residents’ GM-ITE^®^ and mental health scores. The number of permitted beds refers to the number of beds officially authorized and operated by a medical institution in accordance with regional medical plans and regulations. The average length of hospital stay refers to the mean number of days that inpatients spend in the hospital from admission to discharge and is used as an indicator of the operational status of the institution and patients’ clinical course. Participants provided information about their resident level, hospital classification (community or university hospital), postgraduate year (PGY-1 or PGY-2), gender, average number of assigned inpatients, night shifts per month, self-study time per day, and duty-hours per week. After completing the GM-ITE^®^, participants were invited to complete a voluntary questionnaire designed by the Japan Association for Medical Education Program (JAMEP). This questionnaire included items related to the residents’ training environment, including average number of assigned inpatients, night shifts per month, self-study time per day (minutes), and duty-hours per week (hours), training guidance system, clinical training support system, and availability of resources such as academic journals (Supplemental 1). The Mini Z 2.0 survey, a validated tool for assessing burnout and workplace conditions among healthcare professionals, was used [21, 22]. A score of 3 or more on the Mini Z 2.0 survey was classified as indicative of burnout symptoms, reflecting a screening result rather than a clinical diagnosis [21, 22]. The Mini-Z 2.0 Japanese version, which has been adapted to reflect the working conditions of healthcare professionals in Japan, was utilized to ensure contextual relevance and applicability [22]. The yearly inpatient volume at clinical training hospitals was categorized as less than 3,000 (4%), 3,000–5,999 (29%), 6,000–9,999 (31%), and 10,000 or more (36%) based on data from the Japanese MHLW for 2022 [23]. The cut-off of < 3,000 yearly inpatient volumes was chosen to align with the current designation criterion for core clinical training hospitals in Japan, which requires a yearly inpatient volume of at least 3,000 inpatients [9, 23]. Using this policy-based threshold to define the Very Low-Volume group enabled us to directly examine its educational validity. The remaining categories (3,000–5,999, 6,000–9,999, and ≥ 10,000 inpatients per year) were defined to reflect higher inpatient volumes while maintaining a reasonably balanced distribution of hospitals in each group, based on the national inpatient volume distribution reported by the MHLW. [23] In this study, these yearly inpatient volume categories were named Very Low-Volume (< 3,000), Low-Volume (3,000–5,999), Moderate-Volume (6,000–9,999), and High-Volume (≥ 10,000). In addition, we conducted sensitivity analyses in which yearly inpatient volume was treated as a continuous variable, using the same multilevel regression framework and covariates as in the primary categorical models to assess whether our findings were robust to alternative model specifications.

### Statistical analysis

All quantitative data analysis was performed using SPSS Statistics for Windows 26.0 (IBM Corp., Armonk, NY, USA) and SAS software, version 9.4 (SAS Institute Inc., Cary, NC, USA), with statistical significance set at *p* < 0.05. Continuous variables were summarized as mean ± standard deviation (SD) for resident-level data. For facility-level data, continuous variables showing skewed distributions were summarized as median with interquartile range (IQR). Independent samples t-tests were used to compare the means of two groups of continuous variables, and χ² tests were used for comparisons of categorical variables. A multilevel multivariable linear regression analysis with random intercept was conducted to investigate the relationship between yearly inpatient volume and residents’ individual GM-ITE^®^ scores, with the GM-ITE^®^ score as the dependent variable. Multilevel multivariable logistic regression analysis was conducted to investigate the relationship between yearly inpatient volume and resident burnout symptoms, with a positive Mini-Z burnout screen as the dependent variable. In the analysis, hospital-level covariates included the number of permitted beds, annual number of ambulances, annual number of outpatients, number of days in hospital (general ward), number of doctors, number of nurses, and annual number of computed tomography (CT) and magnetic resonance imaging (MRI) scans. Resident-level covariates included postgraduate year (PGY-1 or PGY-2), gender, average number of assigned inpatients, night shifts per month, self-study time per day, and duty-hours per week. These covariates were selected a priori based on previous studies of GM-ITE^®^ performance and resident working conditions, as well as their conceptual relevance as potential confounders of the association between yearly inpatient volume and educational outcomes. To examine potential multicollinearity among hospital-level covariates, variance inflation factors (VIFs) were calculated for the main multilevel models. In addition, we conducted sensitivity analyses in which hospital-level structural variables showing high VIFs (for example, number of permitted beds, annual number of ambulances, annual number of outpatients, number of doctors, and number of nurses) were removed from the models. These models were compared with the primary models to evaluate the robustness of the findings. Resident-level covariates with missing values were treated as missing, and the primary multilevel analyses were conducted as complete-case analyses. As a sensitivity analysis, we re-estimated the multilevel models after including “Unknown” responses as separate indicator categories. Multiple imputation was not performed because the proportion of missing data was small and the pattern of missingness appeared approximately random.

### Ethics statement

This study was conducted in accordance with the principles of the Declaration of Helsinki, and the “Ethical Guidelines for Medical and Health Research Involving Human Subjects.” The research protocol was approved by the JAMEP Ethics Review Committee on December 21, 2023 (approval number: 23 − 12). All participants provided voluntary informed consent and were also given the option to withdraw from the study.

## Results

### Demographics

Overall, 7,498 residents from 608 community-based hospitals participated in the GM-ITE^®^, of which 4,867 residents from 564 facilities consented to participate and were included in the final analysis (response rate: 65.0%). Among all residents, 124, 1,070, 1,850, and 4,074 were affiliated with Very Low-, Low-, Moderate-, and High-Volume hospitals, respectively. In the final analysis, 89 (71.8%), 719 (67.2%), 1,257 (67.9%), and 2,802 (68.8%) residents from these groups were included. Response rates did not differ significantly across inpatient volume categories, including Very Low-Volume hospitals (*p* = 0.612). In Japan, there were 1,028 core clinical training hospitals in 2023, of which 45 were classified as Very Low-Volume, 294 as Low-Volume, 319 as Moderate-Volume, and 370 as High-Volume hospitals [23]. Our study included 25 Very Low-Volume hospitals (55.6% of all Very Low-Volume hospitals), 160 Low-Volume hospitals (54.4%), 176 Moderate-Volume hospitals (55.2%), and 203 High-Volume hospitals (54.9%). The distribution of yearly inpatient volume categories in our sample did not significantly differ from the nationwide distribution (*p* = 0.999). The residents’ characteristics are shown in Table 1. Of the participants, 2,557 (52.5%) were PGY-1 residents, and 1,555 (32.0%) were female. Half of the participants (52.5%) reported being assigned an average of 5–9 inpatients, and 36.9% reported being assigned an average of 0–4 patients. The majority of participants (74.6%) had 3–5 night shifts per month, 10.3% had 6 or more, and 1.8% had none. The self-study time per day was 0 min for 2.7%, 1–30 min for 41.2%, and 31–60 min for 41.8%. More than half of the participants (54.4%) reported working less than 60 duty-hours per week (hours), and 14.6% reported working more than 80 h. Participants’ mean GM-ITE^®^ score was 45.6 (SD: 6.9). Of the participants, 12.4% had burnout symptoms.

**Table 1:** Baseline characteristics of the resident physicians

Resident-level information	Total (*N* = 4867)	High- Volume Hospital (*N* = 2802)	Moderate- Volume Hospital (*N* = 1257)	Low- Volume Hospital (*N* = 719)	Very Low- Volume Hospital (*N* = 89)	*p*-value
Grade						*p* = 0.910
PGY-1, n (%)	2557 (52.5)	1479 (52.8)	650 (51.7)	382 (53.1)	46 (51.7)	
PGY-2, n (%)	2310 (47.5)	1323 (47.2)	607 (48.3)	337 (46.9)	43 (48.3)	
Gender						*p* = 0.024
Men, n (%)	3312 (68.1)	1873 (66.9)	856 (68.1)	514 (71.5)	69 (77.5)	
Women, n (%)	1555 (32.0)	929 (31.2)	401 (31.9)	205 (28.5)	20 (22.5)	
Average number of assigned inpatients						*p* < 0.001
0–4, n (%)	1798 (36.9)	924 (33.0)	534 (42.5)	289 (40.2)	51 (57.3)	
5–9, n (%)	2553 (52.5)	1581 (56.4)	586 (46.6)	356 (49.5)	30 (33.7)	
10–14, n (%)	294 (6.0)	170 (6.1)	76 (6.1)	44 (6.1)	4 (4.5)	
≥ 15, n (%)	113 (2.3)	78 (2.8)	28 (2.2)	6 (0.8)	1 (1.1)	
Unknown, n (%)	109 (2.2)	49 (1.8)	33 (2.6)	24 (3.3)	3 (3.4)	
Night shifts per month						*p* < 0.001
0, n (%)	87 (1.8)	30 (1.1)	22 (1.8)	32 (4.5)	3 (3.4)	
1–2, n (%)	640 (13.2)	378 (13.5)	127 (10.1)	105 (14.6)	30 (33.7)	
3–5, n (%)	3630 (74.6)	2076 (74.1)	950 (75.6)	548 (76.2)	56 (62.9)	
≥ 6, n (%)	502 (10.3)	314 (11.2)	156 (12.4)	32 (4.5)	0 (0.0)	
Unknown, n (%)	8 (0.2)	4 (0.1)	2 (0.2)	2 (0.3)	0 (0.0)	
Self-study time per day (minutes)						*p* = 0.007
0, n (%)	132 (2.7)	1102 (39.3)	556 (44.2)	306 (42.6)	42 (47.2)	
1–30, n (%)	2006 (41.2)	1229 (43.9)	473 (37.6)	298 (41.5)	32 (35.6)	
31–60, n (%)	2032 (41.8)	338 (12.1)	162 (12.9)	72 (10.0)	9 (10.1)	
61–90, n (%)	581 (11.9)	70 (2.5)	26 (2.1)	19 (2.6)	1 (1.1)	
≥ 91, n (%)	116 (2.4)	63 (2.3)	40 (3.2)	24 (3.3)	5 (5.6)	
Duty-hours per week (hours)						*p* < 0.001
Category 1 (< 60), n (%)	2646 (54.4)	1425 (50.9)	699 (55.6)	460 (64.0)	62 (69.7)	
Category 2 (60–79), n (%)	1510 (31.0)	917 (32.7)	394 (31.3)	182 (25.3)	17 (19.1)	
Category 3 (≥ 80), n (%)	711 (14.6)	460 (16.4)	164 (13.1)	77 (10.7)	10 (11.2)	
GM-ITE^®^ score (Mean, SD)	45.6 ± 6.9	46.2 ± 6.9	44.9 ± 6.7	44.3 ± 7.1	45.8 ± 7.3	*p* < 0.001
Burnout symptoms, n (%)	605 (12.4)	338 (12.1)	161 (12.8)	94 (13.1)	12 (13.5)	*p* = 0.831

Table 2 provides facility-level information. The median GM-ITE® score for each facility was 45.0 (IQR: 42.3-47.5). The median burnout symptoms rate for participants at each facility was 7.3% (IQR: 0.0-20.0).

**Table 2:** Background characteristics of the teaching hospitals

Hospital-level information	Total (*N* = 564)	High- Volume Hospital (*N* = 203)	Moderate- Volume Hospital (*N* = 176)	Low- Volume Hospital (*N* = 160)	Very Low- Volume Hospital (*N* = 25)	*p*-value
Number of permitted beds (×100), Median (IQR)	4.0 (3.1-5.0)	5.4 (4.8–6.4)	3.8 (3.4–4.1)	2.8 (2.5–3.2)	2.0 (1.6–3.3)	*p* < 0.001
Annual number of ambulances (×100), Median (IQR)	12.7 (7.9–19.7)	20.9 (16.0-26.7)	11.4 (8.6–16.0)	7.5 (5.3–10.8)	4.5 (2.0-7.1)	*p* < 0.001
Annual number of outpatients (×100), Median (IQR)	6.5 (4.5–8.7)	9.4 (7.5–11.7)	6.4 (5.0-7.8)	4.6 (3.3–5.8)	2.9 (2.2–4.5)	*p* = 0.004
Number of days in hospital, Median (IQR)	12.8 (11.3–14.8)	12.0 (10.7–13.2)	13.2 (11.6–15.1)	13.7 (12.0–16.0)	14.5 (12.8–17.3)	*p* < 0.001
Number of doctors (×100), Median (IQR)	0.9 (0.6–1.4)	1.5 (1.2-2.0)	0.8 (0.7-1.0)	0.6 (0.4–0.7)	0.4 (0.3–0.5)	*p* < 0.001
Number of nurses (×100), Median (IQR)	3.4 (2.5–4.8)	5.6 (4.5–6.5)	3.3 (2.9–3.8)	2.4 (2.0-2.9)	1.7 (1.2–2.2)	*p* < 0.001
Annual number of CT scans (×1000), Median (IQR)	15.2 (9.5–22.5)	24.3 (18.0-30.5)	15.0 (10.9–18.5)	9.7 (7.2–12.7)	5.8 (4.5–9.2)	*p* < 0.001
Annual number of MRI scans (×1000), Median (IQR)	5.1 (3.2–7.6)	7.7 (6.0-10.1)	4.9 (3.3–6.8)	3.2 (2.3–4.3)	1.8 (1.4–3.7)	*p* < 0.001
GM-ITE^®^ score, Median (IQR)	45.0 (42.3–47.5)	45.9 (44.1–47.6)	45.0 (42.1–47.5)	44.0 (41.1–46.8)	45.1 (42.0–49.0)	*p* < 0.001
Burnout symptoms, Median (IQR), %	7.3 (0.0–20.0)	9.5 (0.0-19.2)	7.4 (0.0-22.2)	0.0 (0.0–20.0)	0.0 (0.0–25.0)	*p* = 0.211

Supplemental 2 presents the relationship between GM-ITE^®^ scores at the hospital level and individual resident level in the univariate analysis. At the hospital level, statistically significant factors included the number of permitted beds, annual number of ambulances, annual number of outpatients, average length of hospital stay (general ward), number of doctors, number of nurses, annual number of CT and MRI scans. At the resident level, statistically significant factors included year of training, average number of assigned inpatients, night shifts per month, self-study time per day, and duty-hours per week. In addition to these factors, gender was included in the multivariate analysis. Table 3 presents the relationship between GM-ITE^®^ scores and hospital- and resident-level information using multivariate analysis. In the multivariate analysis, the GM-ITE^®^ score was significantly higher in the Very Low-Volume group than in the Low-Volume group (coefficient: 2.007; 95% confidence interval (CI): 0.298–3.717, *p* = 0.021). However, the GM-ITE^®^ scores of the Very Low-Volume group did not differ significantly from those of the Moderate-Volume (coefficient: 1.637; 95% CI: -0.070–3.344, *p* = 0.060) and High-Volume (coefficient: 1.638; 95% CI: -0.206–3.482, *p* = 0.082) groups.

**Table 3:** The relationship between GM-ITE^®^ scores and hospital- and resident-level information using multilevel analysis

Factors	Adjusted estimated coefficient (95% CI)	*p*-value
Average number of inpatients		
Hospital-level information		
Very Low-Volume Hospitals	Reference	Reference
Low-Volume Hospitals	-2.007 (-3.717 to -0.298)	*p* = 0.021
Moderate-Volume Hospitals	-1.637 (-3.344 to 0.070)	*p* = 0.060
High-Volume Hospitals	-1.638 (-3.482 to 0.206)	*p* = 0.082
Number of permitted beds	-0.147 (-0.469 to 0.175)	*p* = 0.371
Annual number of ambulances	0.019 (-0.027 to 0.066)	*p* = 0.416
Annual number of outpatients	0.063 (-0.043 to 0.169)	*p* = 0.246
Number of days in hospital	-0.011 (-0.055 to 0.033)	*p* = 0.613
Number of doctors	0.727 (-0.116 to 1.570)	*p* = 0.091
Number of nurses	0.298 (-0.032 to 0.628)	*p* = 0.077
Annual number of CT scans	-0.060 (-0.097 to -0.022)	*p* = 0.002
Annual number of MRI scans	0.042 (-0.020 to 0.104)	*p* = 0.186
Resident-level information		
Grade		
PGY-1	Reference	Reference
PGY-2	1.667 (1.289 to 2.045)	*p* < 0.001
Gender		
Men	Reference	Reference
Women	0.015 (-0.388 to 0.419)	*p* = 0.940
Average number of assigned inpatients		
0–4	Reference	Reference
5–9	1.070 (0.640 to 1.501)	*p* < 0.001
10–14	0.857 (-0.023 to 1.738)	*p* = 0.056
≥ 15	0.508 (-0.958 to 1.974)	*p* = 0.497
Unknown	0.409 (-0.893 to 1.712)	*p* = 0.538
Night shifts per month		
0	Reference	Reference
1–2	0.384 (-1.178 to 1.944)	*p* = 0.630
3–5	0.568 (-0.919 to 2.054)	*p* = 0.454
≥ 6	0.414 (-1.222 to 2.049)	*p* = 0.620
Unknown	3.031 (-1.770 to 7.833)	*p* = 0.216
Self-study time per day (minutes)		
1–30	Reference	Reference
31–60	0.690 (0.276 to 1.103)	*p* = 0.001
61–90	1.192 (0.566 to 1.818)	*p* < 0.001
≥ 91	1.368 (0.120 to 2.615)	*p* = 0.032
0	-1.252 (-2.422 to -0.083)	*p* = 0.036
Duty-hours per week (hours)		
Category 1 (< 60), n (%)	Reference	Reference
Category 2 (60–79), n (%)	0.592 (0.156 to 1.028)	*p* = 0.008
Category 3 (≥ 80), n (%)	-0.128 (-0.711 to 0.455)	*p* = 0.667

Supplemental 3 presents the relationship between hospital-level and resident-level information and burnout symptoms in the unadjusted logistic regression analysis. At the hospital level, no statistically significant factors were identified. At the resident level, the statistically significant factors included the average number of assigned inpatients and the number of night shifts per month. In the multivariable analysis, adjustments were made for hospital-level factors, including the number of beds, annual number of ambulances, annual number of outpatients, number of days in hospital, number of doctors, number of nurses, annual number of CT and MRI scans, as well as resident-level factors, including year of training, gender, night shifts per month, and self-study time per day. The multivariable analysis revealed no significant differences in burnout symptoms between the Very Low-Volume group and the Low-Volume (adjusted odds ratio (aOR): 0.974; 95% CI: 0.493–1.925, *p* = 0.940), Moderate-Volume (aOR: 0.967; 95% CI: 0.492–1.903, *p* = 0.923), or High-Volume (aOR: 0.994; 95% CI: 0.485–2.034, *p* = 0.986) groups (Table 4).

**Table 4:** The relationship between burnout in residents and hospital- and resident-level information using multilevel analysis

Factors	Adjusted odds ratio (95% CI)	*p*-value
Average number of inpatients		
Hospital-level information		
Very Low-Volume Hospitals	Reference	Reference
Low-Volume Hospitals	0.974 (0.493 to 1.925)	*p* = 0.940
Moderate-Volume Hospitals	0.967 (0.492 to 1.903)	*p* = 0.923
High-Volume Hospitals	0.994 (0.485 to 2.034)	*p* = 0.986
Number of permitted beds	0.962 (0.856 to 1.083)	*p* = 0.523
Annual number of ambulances	1.021 (1.004 to 1.039)	*p* = 0.017
Annual number of outpatients	1.026 (0.988 to 1.065)	*p* = 0.177
Number of days in hospital	1.000 (0.982 to 1.020)	*p* = 0.962
Number of doctors	1.145 (0.849 to 1.543)	*p* = 0.373
Number of nurses	0.918 (0.810 to 1.040)	*p* = 0.176
Annual number of CT scans	0.995 (0.982 to 1.008)	*p* = 0.447
Annual number of MRI scans	1.002 (0.978 to 1.027)	*p* = 0.855
Resident-level information		
Grade		
PGY-1	Reference	Reference
PGY-2	1.045 (0.878 to 1.243)	*p* = 0.620
Gender		
Men	Reference	Reference
Women	1.133 (0.937 to 1.370)	*p* = 0.197
Average number of assigned inpatients		
0–4	Reference	Reference
5–9	1.040 (0.856 to 1.263)	*p* = 0.692
10–14	0.794 (0.551 to 1.144)	*p* = 0.215
≥ 15	0.441 (0.266 to 0.731)	*p* = 0.002
Unknown	1.114 (0.603 to 2.067)	*p* = 0.730
Night shifts per month		
0	Reference	Reference
1–2	1.077 (0.548 to 2.117)	*p* = 0.830
3–5	1.237 (0.651 to 2.350)	*p* = 0.517
≥ 6	1.249 (0.620 to 2.515)	*p* = 0.534
Unknown	0.496 (0.085 to 2.894)	*p* = 0.436
Self-study time per day (minutes)		
1–30	Reference	Reference
31–60	1.128 (0.931 to 1.366)	*p* = 0.219
61–90	1.126 (0.844 to 1.501)	*p* = 0.419
≥ 91	1.285 (0.703 to 2.348)	*p* = 0.415
0	0.880 (0.535 to 1.446)	*p* = 0.613
Duty-hours per week (hours)		
Category 1 (< 60), n (%)	Reference	Reference
Category 2 (60–79), n (%)	0.786 (0.643 to 0.962)	*p* = 0.019
Category 3 (≥ 80), n (%)	0.646 (0.501 to 0.832)	*p* < 0.001

Multicollinearity diagnostics showed that several hospital-level structural variables exhibited elevated VIFs, consistent with their representing the same underlying construct of hospital size and resource availability. In sensitivity analyses excluding these highly collinear variables, the estimated associations between yearly inpatient volume and both GM-ITE^®^ scores and burnout symptoms remained similar in direction and magnitude to those of the main models, and the overall conclusions were unchanged (Supplemental 4, 5, 6 and 7). In the sensitivity analyses that included “Unknown” responses as separate categories, the estimates for yearly inpatient volume and both outcomes were essentially unchanged in direction and magnitude compared with the primary complete-case models.

In further sensitivity analyses treating yearly inpatient volume as a continuous variable (Supplemental 8 and 9), inpatient volume was not significantly associated with either GM-ITE^®^ scores or burnout symptoms, and the effect estimates were similar to those of the categorical models, indicating that the main findings were robust to this alternative specification.

The relationship between yearly inpatient volume and information on the training environment for residents (training guidance system, clinical training support system, and academic journal availability) is presented in Supplemental 10, 11, and 12. For the training guidance system and clinical training support system, the proportion of residents who selected “Strongly disagree” was higher, and the proportion of residents who selected “Strongly agree” was lower in the Very Low-Volume group than in the other groups. Similarly, for academic journal availability, the proportion of residents who selected “Strongly agree” was lower in the Very Low-Volume group than in the other groups.

## Discussion

The results of this study provide an important resource for policymaking related to the education of medical residents in Japan. In the multivariate analysis of GM-ITE^®^ scores, the Very Low-Volume group had significantly higher GM-ITE^®^ scores than those of the Low-Volume group. The adjusted difference was approximately 2 points on an 80-point scale, indicating a small effect size at the level of individual residents; however, even such modest differences in mean scores may have educational relevance when comparing training environments and considering criteria for hospital accreditation. In contrast, no significant differences were observed between the Very Low-Volume group and the Moderate-Volume or High-Volume groups. In the multivariable analysis of burnout symptoms among residents, the prevalence of a positive Mini-Z burnout screen did not differ significantly between the Very Low-Volume group and the Low-Volume, Moderate-Volume, or High-Volume groups. This suggests that Very Low-Volume hospitals may provide an educational environment in which basic clinical knowledge can be acquired without clear disadvantage compared with larger hospitals, and accreditation decisions need not be based solely on inpatient volume when other aspects of the educational environment are demonstrated to be adequate. Taken together, these results are more consistent with non-inferiority or absence of disadvantage of Very Low-Volume hospitals, rather than demonstrating their superiority. In addition, the cross-sectional design means that residual and unmeasured confounding (for example, residents’ prior academic performance or intrinsic motivation) may partly explain the observed associations. These interpretations were supported by sensitivity analyses using inpatient volume as a continuous variable, which likewise did not demonstrate significant associations with GM-ITE^®^ scores or burnout symptoms.

However, analysis of the effect of yearly inpatient volume on the training guidance system, clinical training support system, and academic journal availability suggests that the training environment for residents in the Very Low-Volume group could be improved, particularly regarding the training guidance and clinical training support systems, and academic journal availability. In the training environment analysis, the Very Low-Volume group showed a consistent pattern in which residents more frequently selected extreme response categories. Evaluations of the training guidance system and academic journal availability tended to include higher proportions of negative assessments and fewer strongly positive assessments compared with other inpatient volume groups. These patterns suggest that, despite comparable GM-ITE^®^ scores and burnout symptoms, certain structural elements of the training environment may be less well developed in Very Low-Volume hospitals. These findings also carry implications for accreditation. Policymakers may need to consider minimum expectations for supervision quality, training support systems, and academic resource availability that are independent of inpatient volume. Targeted interventions—such as improving access to online journals, strengthening administrative support for training programs, and promoting inter-hospital educational collaboration—may help address these limitations and further enhance training quality.

In hospitals with a large number of inpatients, sufficient numbers of supervising physicians and nurses allow residents to gain experience in patient care, including interprofessional collaboration. Conversely, a shortage of supervising physicians and nurses might impose excessive burdens on residents and adversely affect their learning opportunities and mental health [7, 11]. Similarly, CT and MRI capacity shapes access to advanced imaging and influences patterns of test utilization. Hospitals with multiple scans can provide more timely access to imaging and expose residents to a broader range of diagnostic indications, whereas limited imaging resources may necessitate more selective use of imaging and greater reliance on clinical assessment when deciding whether to order tests [24, 25]. A comprehensive evaluation of these factors is essential to improve the effectiveness of resident medical education and enhance the quality of patient care.

### Comparison with previous studies on the number of inpatients and GM-ITE^®^ scores of residents

Previous studies have demonstrated a positive correlation between the number of inpatients and the GM-ITE^®^ scores of residents [26]. The advantages of clinical training at high-volume hospitals include a wide range of specialist departments, providing residents with opportunities to gain experience with specialized medical care and intensive care, including tertiary emergency care and the management of critically ill patients. High-volume hospitals also tend to offer abundant opportunities for research and academic activities and have well-organized, systematic clinical training programs. Furthermore, frequent encounters with inpatients are crucial for improving basic clinical skills. However, the quality of patient care should also be considered. Teaching hospitals, regardless of inpatient volume, are generally capable of providing high-quality care, which can also enhance the quality of education [26–28]. The case load can affect the quality of care provided [29, 30]. 

### Advantages of clinical training at very Low-Volume hospitals

In the multivariate analysis, the GM-ITE^®^ score of the Very Low-Volume group was significantly higher than that of the Low-Volume group. However, in the multivariable logistic regression analysis, burnout symptoms did not differ significantly according to the yearly inpatient volume. Clinical training at Very Low-Volume hospitals can improve patient care ownership [31–34]. In Very Low-Volume hospitals, residents are often allowed to take an active role in providing comprehensive medical care, taking responsibility for the overall care of patients from general outpatient care, hospitalization, treatment, to follow-up after discharge [31, 32]. Notably, patient care ownership, which emphasizes residents’ active engagement in patient care, has been identified as a critical component of medical training that fosters professional growth and responsibility in Japan as well [35]. The framework for patient care ownership within Japan’s unique clinical training environment highlights the importance of integrating residents into the continuity of care, enabling them to better understand their role in patient outcomes [35]. Although the team approach is strictly enforced in High-Volume Hospitals and the roles of each specialty tend to be divided, in Very Low-Volume Hospitals, residents are often required to make decisions on their own [31, 32]. This may promote the development of clinical judgment and clinical leadership [31, 32]. Another advantage of clinical training in Very Low-Volume Hospitals is that it provides community-based education [36, 37]. Very Low-Volume hospitals are closely connected to the local community, enabling residents to acquire practical knowledge of community healthcare. In addition, community-based healthcare delivery systems provide opportunities for active participation in interprofessional collaboration in situations such as the community-based integrated care system [36, 37]. In addition to patient care ownership, and community-based education, advantages of training in small hospitals may include receiving customized education that suits residents’ individual needs, having opportunities to see patients in a wide range of fields without specializing in a particular field, and being close to supervisors. The results of this study suggest that residents working in Very Low-Volume Hospitals can acquire basic clinical skills with no evidence of worse GM-ITE^®^ scores or burnout symptoms compared with residents in larger hospitals.

### Disadvantages of clinical training at very Low-Volume hospitals

The results of the relationship between the yearly inpatient volume and the training environment for residents suggest that the training environment in Very Low-Volume Hospitals could be improved. Although having an adequate number of supervisors is important, residents also need access to physicians from a range of disciplines with whom they can consult [38–43]. Such support is essential for improving the training environment and maintaining the mental health of residents [38–43]. Among these roles, clinical training administrative staff play a crucial educational role for residents [38]. Their responsibilities include coordinating and liaising with various hospital departments, enhancing the training environment, supporting residents, and providing adult education [38]. In addition to handling paperwork, clinical training administrative staff often act as mentors who require skill development and institutional support [38]. Enhancing the competencies of clinical training administrative staff could contribute significantly to improving the training environment for residents at Very Low-Volume Hospitals.

### Recommendations for improving the quality of clinical training based on this research

The results of this study align with the accreditation standards of the Accreditation Council for Graduate Medical Education (ACGME), which emphasizes the quality of educational programs from an international perspective [44]. These findings may offer useful context for understanding how inpatient volume relates to international clinical training programs [44]. This study indicates that residents working in Very Low-Volume Hospitals develop basic clinical skills comparable to those of residents in High-Volume Hospitals. However, there is room for improvement in the clinical training environment at Very Low-Volume Hospitals. Enhancing the clinical training environment to support residents’ growth and career development, such as through the strengthening of administrative staff responsible for clinical training, is critical. Because we did not assess key accreditation factors such as supervision quality, rotation availability, or resident support systems, our results should be viewed as evidence on inpatient volume rather than as sufficient grounds for accreditation decisions. Such efforts are essential for further improving the quality of clinical training at Very Low-Volume Hospitals.

### Limitations

This study has several limitations. First, the data used in this study were primarily self-reported at the end of the GM-ITE^®^. Although self-reported data provides a comprehensive overview, it may include biases and inaccuracies, leading to potential discrepancies between reported and actual experience. This study was conducted exclusively with residents working in community hospitals in Japan; therefore, these findings may not be generalizable to university hospitals or to other countries. Although this study offers valuable insights into the Japanese medical education system, further studies are required to determine whether these results can be applied to medical residents in other countries. Third, this study may have selection bias because it included only participants from the voluntary GM-ITE^®^ program. The participants accounted for approximately one-third of all residents in Japan, and the training hospitals that participated were likely those with a strong focus on education. Furthermore, we lacked data on hospitals that did not participate in the GM-ITE^®^, so selection bias at the hospital level cannot be excluded. Within participating community-based hospitals, however, response rates were similar across inpatient volume categories (including Very Low-Volume hospitals), which partly reduces concerns about differential selection by inpatient volume. Fourth, the study was conducted at a single timepoint and did not consider the evolution of clinical training programs. This survey aimed to explore the relationship between the yearly inpatient volume at clinical training hospitals, GM-ITE^®^ scores, and resident burnout symptoms, presenting results that reflect a specific point in time. These findings may evolve in response to future feedback and policy changes. Fifth, although we adjusted for several hospital-level and resident-level characteristics, we could not account for all potential confounding variables (for example, residents’ prior academic performance, intrinsic motivation, or informal support), so residual and unmeasured confounding may have influenced the associations between yearly inpatient volume, GM-ITE^®^ scores, and burnout symptoms. In particular, we lacked rotation- or ward-level workload indicators, such as patient-to-physician ratios in specific services (e.g. internal medicine, emergency medicine, pediatrics), so important differences in team-level workload across hospitals may not have been fully captured by our hospital-level variables. In addition, some resident-level covariates contained missing values; although the proportion of missing data was small and the distribution of “Unknown” responses did not differ markedly across yearly inpatient volume categories, we cannot exclude the possibility that non-random missingness may have introduced bias. Sixth, although we additionally modeled yearly inpatient volume as a continuous variable in sensitivity analyses, these models also showed no significant association with GM-ITE^®^ scores or burnout symptoms; nevertheless, we cannot exclude the possibility of nonlinear effects or residual confounding that might not be fully captured by our models. Seventh, the Mini-Z 2.0 survey used a single-item question to assess burnout symptoms. Although this single-item measure has high specificity for identifying a positive burnout screen, it is not equivalent to a clinical diagnosis of burnout, and its limited sensitivity may have led to an underestimation of residents experiencing burnout symptoms [45, 46]. The apparent reversal in the burnout analysis likely reflects a statistical suppression effect from adjusting for correlated workload- and hospital-level covariates rather than true model instability. Because the underlying association was weak and the adjusted odds ratios remained close to 1.0 across all models, this reversal is best interpreted as a statistical artifact rather than a meaningful contradictory effect. Finally, the response rate of 65.0% may have affected the representativeness of the results.

## Conclusions

This study shows that residents at Very Low-Volume Hospitals achieve GM-ITE^®^ scores and mental health outcomes comparable to those at larger hospitals. Although some structural aspects may require improvement, these hospitals can support resident education and well-being when appropriate standards are met; still, accreditation decisions must consider factors such as supervision quality, rotation availability, and support systems that were not fully assessed here. The consistency of categorical and continuous-volume analyses indicates that inpatient volume alone was not meaningfully associated with resident performance or well-being.

## Supplementary Information

Supplementary Material 1.

Supplementary Material 2.

Supplementary Material 3.

Supplementary Material 4.

Supplementary Material 5.

Supplementary Material 6.

Supplementary Material 7.

Supplementary Material 8.

Supplementary Material 9.

Supplementary Material 10.

Supplementary Material 11.

Supplementary Material 12.

## Data Availability

Resident physicians who joined in this study did not obtain consent for their data to be shared publicly, so supporting data is not available. The corresponding author will respond to inquiries on the data analyses in this study.
