# Supplementary material for: Impact of inpatient volume on residents’ In-training examination scores and burnout in Japanese community hospitals: a nationwide cross-sectional study
Source: BMC Med Educ. 2026 Jan 24;26:409. doi: 10.1186/s12909-026-08664-3 (PMC12980981; doi:10.1186/s12909-026-08664-3)
Supplement: Supplementary file 1 — Supplementary Material 1. [file 12909_2026_8664_MOESM1_ESM.docx]

**Supplemental 1:** Questionnaire about residents' training environment.

**1. Average number of assigned inpatients**

(1) 0-4

(2) 5-9

(3) 10-14

(4) ≥ 15

(5) Unknown

**2. Average night shifts per month**

(1) 0

(2) 1-2

(3) 3-5

(4) ≥ 6

(5) Unknown

**3. Average self-study time per day (minutes)**

(1) 1-30

(2) 31-60

(3) 61-90

(4) ≥ 91

(5) None

**4. Duty-hours per week (hours)**

(1) < 60

(2) 60-79

(3) ≥ 80

**5. Are you satisfied with the training guidance system at your clinical training hospital?**

(1) Strongly disagree

(2) Disagree

(3) Neither agree nor disagree

(4) Agree

(5) Strongly agree

**6. Do you think the clinical training support system at your clinical training hospital is well-developed for training purposes?**

(1) Strongly disagree

(2) Disagree

(3) Neither agree nor disagree

(4) Agree

(5) Strongly agree

**7. Do you think academic journal availability at your clinical training hospital is sufficient for training purposes?**

(1) Strongly disagree

(2) Disagree

(3) Neither agree nor disagree

(4) Agree

(5) Strongly agree
