# Supplementary material for: Impact of inpatient volume on residents’ In-training examination scores and burnout in Japanese community hospitals: a nationwide cross-sectional study
Source: BMC Med Educ. 2026 Jan 24;26:409. doi: 10.1186/s12909-026-08664-3 (PMC12980981; doi:10.1186/s12909-026-08664-3)
Supplement: Supplementary file 2 — Supplementary Material 2. [file 12909_2026_8664_MOESM2_ESM.docx]

**Supplemental 2:** The relationship between GM-ITE^®^ scores and hospital- and resident-level information using univariate analysis.

| **Factors** | Estimated coefficient (95% CI) | p-value |
| --- | --- | --- |
| **Hospital-level information** |  |  |
| **Average number of inpatients** |  |  |
| Very Low- Volume Hospitals | Reference | Reference |
| Low- Volume Hospitals | -1.510 (-3.021 to 0.001) | p = 0.050 |
| Moderate- Volume Hospitals | -0.855 (-2.330 to 0.620) | p = 0.256 |
| High- Volume Hospitals | 0.381 (-1.067 to 1.828) | p = 0.606 |
| **Number of permitted beds** | 0.434 (0.331 to 0.537) | p < 0.001 |
| **Annual number of ambulances** | 0.064 (0.044 to 0.085) | p < 0.001 |
| **Annual number of outpatients** | 0.164 (0.120 to 0.208) | p < 0.001 |
| **Number of days in hospital** | -0.046 (-0.085 to -0.008) | p = 0.019 |
| **Number of doctors** | 1.081 (0.852 to 1.309) | p < 0.001 |
| **Number of nurses** | 0.429 (0.341 to 0.516) | p < 0.001 |
| **Annual number of CT scans** | 0.027 (0.013 to 0.042) | p < 0.001 |
| **Annual number of MRI scans** | 0.085 (0.049 to 0.121) | p < 0.001 |
| **Resident-level information** |  |  |
| **Grade** |  |  |
| PGY-1 | Reference | Reference |
| PGY-2 | 1.764 (1.379 to 2.149) | p < 0.001 |
| **Gender** |  |  |
| Men | Reference | Reference |
| Women | -0.182 (-0.598 to 0.233) | p = 0.390 |
| **Average number of assigned inpatients** |  |  |
| 0-4 | Reference | Reference |
| 5-9 | 1.511 (1.097 to 1.926) | p < 0.001 |
| 10-14 | 1.471 (0.625 to 2.318) | p = 0.001 |
| ≥ 15 | 0.836 (-0.468 to 2.141) | p = 0.209 |
| Unknown | 0.176 (-1.151 to 1.503) | p = 0.795 |
| **Night shifts per month** |  |  |
| 0 | Reference | Reference |
| 1-2 | 1.015 (-0.530 to 2.559) | p = 0.198 |
| 3-5 | 1.568 (0.102 to 3.034) | p = 0.036 |
| ≥ 6 | 1.453 (-0.117 to 3.022) | p = 0.070 |
| Unknown | 3.260 (-1.733 to 8.254) | p = 0.201 |
| **Self-study time per day (minutes)** |  |  |
| 1-30 | Reference | Reference |
| 31-60 | 1.240 (0.818 to 1.662) | p < 0.001 |
| 61-90 | 2.144 (1.512 to 2.776) | p < 0.001 |
| ≥ 91 | 2.187 (0.905 to 3.469) | p = 0.001 |
| 0 | -1.469 (-2.675 to -0.263) | p = 0.017 |
| **Duty-hours per week (hours)** |  |  |
| Category 1 (< 60), n (%) | Reference | Reference |
| Category 2 (60–79), n (%) | 1.037 (0.602 to 1.473) | p < 0.001 |
| Category 3 (≥ 80), n (%) | 0.475 (-0.095 to 1.045) | p = 0.102 |
