# Supplementary material for: Impact of inpatient volume on residents’ In-training examination scores and burnout in Japanese community hospitals: a nationwide cross-sectional study
Source: BMC Med Educ. 2026 Jan 24;26:409. doi: 10.1186/s12909-026-08664-3 (PMC12980981; doi:10.1186/s12909-026-08664-3)
Supplement: Supplementary file 3 — Supplementary Material 3. [file 12909_2026_8664_MOESM3_ESM.docx]

**Supplemental 3:** The relationship between burnout symptoms in residents and hospital- and resident-level information using univariate analysis.

| **Factors** | Odds ratio (95% CI) | p-value |
| --- | --- | --- |
| **Hospital-level information** |  |  |
| **Average number of inpatients** |  |  |
| Very Low-Volume Hospitals | Reference | Reference |
| Low-Volume Hospitals | 0.977 (0.513 to 1.862) | p = 0.944 |
| Moderate-Volume Hospitals | 0.936 (0.498 to 1.757) | p = 0.836 |
| High-Volume Hospitals | 0.876 (0.472 to 1.626) | p = 0.675 |
| **Number of permitted beds** | 0.986 (0.942 to 1.032) | p = 0.551 |
| **Annual number of ambulances** | 0.992 (0.984 to 1.001) | p = 0.098 |
| **Annual number of outpatients** | 0.989 (0.970 to 1.009) | p = 0.267 |
| **Number of days in hospital** | 1.004 (0.990 to 1.019) | p = 0.539 |
| **Number of doctors** | 0.958 (0.865 to 1.062) | p = 0.416 |
| **Number of nurses** | 0.989 (0.952 to 1.029) | p = 0.594 |
| **Annual number of CT scans** | 0.999 (0.992 to 1.005) | p = 0.656 |
| **Annual number of MRI scans** | 0.992 (0.975 to 1.009) | p = 0.349 |
| **Resident-level information** |  |  |
| **Grade** |  |  |
| PGY-1 | Reference | Reference |
| PGY-2 | 0.967 (0.816 to 1.145) | p = 0.694 |
| **Gender** |  |  |
| Men | Reference | Reference |
| Women | 0.882 (0.733 to 1.061) | p = 0.182 |
| **Average number of assigned inpatients** |  |  |
| 0-4 | Reference | Reference |
| 5-9 | 0.956 (0.795 to 1.150) | p = 0.631 |
| 10-14 | 1.321 (0.936 to 1.863) | p = 0.113 |
| ≥ 15 | 2.439 (1.575 to 3.775) | p < 0.001 |
| Unknown | 0.970 (0.534 to 1.759) | p = 0.919 |
| **Night shifts per month** |  |  |
| 0 | Reference | Reference |
| 1-2 | 0.891 (0.464 to 1.711) | p = 0.730 |
| 3-5 | 0.866 (0.467 to 1.606) | p = 0.649 |
| ≥ 6 | 0.976 (0.504 to 1.888) | p = 0.942 |
| Unknown | 2.084 (0.376 to 11.547) | p = 0.401 |
| **Self-study time per day (minutes)** |  |  |
| 1-30 | Reference | Reference |
| 31-60 | 0.892 (0.740 to 1.075) | p = 0.229 |
| 61-90 | 0.948 (0.719 to 1.251) | p = 0.707 |
| ≥ 91 | 0.832 (0.461 to 1.501) | p = 0.540 |
| 0 | 1.248 (0.770 to 2.024) | p = 0.368 |
| **Duty-hours per week (hours)** |  |  |
| Category 1 (< 60), n (%) | Reference | Reference |
| Category 2 (60–79), n (%) | 1.257 (1.038 to 1.522) | p = 0.019 |
| Category 3 (≥ 80), n (%) | 1.579 (1.252 to 1.991) | p < 0.001 |
