# Supplementary material for: Impact of inpatient volume on residents’ In-training examination scores and burnout in Japanese community hospitals: a nationwide cross-sectional study
Source: BMC Med Educ. 2026 Jan 24;26:409. doi: 10.1186/s12909-026-08664-3 (PMC12980981; doi:10.1186/s12909-026-08664-3)
Supplement: Supplementary file 4 — Supplementary Material 4. [file 12909_2026_8664_MOESM4_ESM.docx]

**Supplemental 4:** Sensitivity analysis: Multilevel model for GM-ITE^®^ scores including yearly inpatient volume and minimally adjusted hospital- and resident-level covariates.

| **Factors** | Adjusted estimated coefficient (95% CI) | p-value |
| --- | --- | --- |
| **Hospital-level information** |  |  |
| **Average number of inpatients** |  |  |
| Very Low-Volume Hospitals | Reference | Reference |
| Low-Volume Hospitals | -1.929 (-3.657 to -0.201) | p = 0.029 |
| Moderate-Volume Hospitals | -1.377 (-3.095 to 0.341) | p = 0.116 |
| High-Volume Hospitals | -1.023 (-2.852 to 0.806) | p = 0.273 |
| **Number of permitted beds** | 0.353 (0.114 to 0.591) | p = 0.004 |
| **Annual number of CT scans** | -0.027 (-0.060 to 0.007) | p = 0.120 |
| **Annual number of MRI scans** | 0.043 (-0.020 to 0.106) | p = 0.179 |
| **Resident-level information** |  |  |
| **Grade** |  |  |
| PGY-1 | Reference | Reference |
| PGY-2 | 1.656 (1.279 to 2.034) | p < 0.001 |
| **Gender** |  |  |
| Men | Reference | Reference |
| Women | 0.046 (-0.358 to 0.450) | p = 0.823 |
| **Average number of assigned inpatients** |  |  |
| 0-4 | Reference | Reference |
| 5-9 | 1.089 (0.658 to 1.520) | p < 0.001 |
| 10-14 | 0.849 (-0.033 to 1.731) | p = 0.059 |
| ≥ 15 | 0.503 (-0.960 to 1.966) | p = 0.501 |
| Unknown | 0.340 (-0.964 to 1.644) | p = 0.609 |
| **Night shifts per month** |  |  |
| 0 | Reference | Reference |
| 1-2 | 0.365 (-1.200 to 1.930) | p = 0.648 |
| 3-5 | 0.509 (-0.983 to 2.000) | p = 0.504 |
| ≥ 6 | 0.345 (-1.295 to 1.985) | p = 0.680 |
| Unknown | 3.035 (-1.769 to 7.840) | p = 0.216 |
| **Self-study time per day (minutes)** |  |  |
| 1-30 | Reference | Reference |
| 31-60 | 0.688 (0.274 to 1.102) | p = 0.001 |
| 61-90 | 1.178 (0.552 to 1.804) | p < 0.001 |
| ≥ 91 | 1.317 (0.069 to 2.566) | p = 0.039 |
| 0 | -1.269 (-2.437 to -0.100) | p = 0.033 |
| **Duty-hours per week (hours)** |  |  |
| Category 1 (< 60), n (%) | Reference | Reference |
| Category 2 (60–79), n (%) | 0.596 (0.160 to 1.033) | p = 0.007 |
| Category 3 (≥ 80), n (%) | -0.100 (-0.682 to 0.482) | p = 0.737 |
