# Supplementary material for: Impact of inpatient volume on residents’ In-training examination scores and burnout in Japanese community hospitals: a nationwide cross-sectional study
Source: BMC Med Educ. 2026 Jan 24;26:409. doi: 10.1186/s12909-026-08664-3 (PMC12980981; doi:10.1186/s12909-026-08664-3)
Supplement: Supplementary file 5 — Supplementary Material 5. [file 12909_2026_8664_MOESM5_ESM.docx]

**Supplemental 5:** Sensitivity analysis: Multilevel model for GM-ITE^®^ scores adjusted for yearly inpatient volume and resident-level covariates only.

| **Factors** | Adjusted estimated coefficient (95% CI) | p-value |
| --- | --- | --- |
| **Hospital-level information** |  |  |
| **Average number of inpatients** |  |  |
| Very Low-Volume Hospitals | Reference | Reference |
| Low-Volume Hospitals | -1.770 (-3.506 to -0.033) | p = 0.046 |
| Moderate-Volume Hospitals | -1.000 (-2.707 to 0.707) | p = 0.251 |
| High-Volume Hospitals | -0.091 (-1.773 to 1.592) | p = 0.916 |
| **Resident-level information** |  |  |
| **Grade** |  |  |
| PGY-1 | Reference | Reference |
| PGY-2 | 1.666 (1.288 to 2.045) | p < 0.001 |
| **Gender** |  |  |
| Men | Reference | Reference |
| Women | 0.053 (-0.351 to 0.457) | p = 0.799 |
| **Average number of assigned inpatients** |  |  |
| 0-4 | Reference | Reference |
| 5-9 | 1.097 (0.665 to 1.529) | p < 0.001 |
| 10-14 | 0.858 (-0.026 to 1.741) | p = 0.057 |
| ≥ 15 | 0.367 (-1.093 to 1.827) | p = 0.622 |
| Unknown | 0.310 (-0.994 to 1.615) | p = 0.641 |
| **Night shifts per month** |  |  |
| 0 | Reference | Reference |
| 1-2 | 0.517 (-1.048 to 2.082) | p = 0.517 |
| 3-5 | 0.603 (-0.890 to 2.096) | p = 0.429 |
| ≥ 6 | 0.402 (-1.241 to 2.044) | p = 0.632 |
| Unknown | 3.115 (-1.691 to 7.921) | p = 0.204 |
| **Self-study time per day (minutes)** |  |  |
| 1-30 | Reference | Reference |
| 31-60 | 0.697 (0.282 to 1.111) | p = 0.001 |
| 61-90 | 1.191 (0.564 to 1.817) | p < 0.001 |
| ≥ 91 | 1.298 (0.049 to 2.547) | p = 0.042 |
| 0 | -1.232 (-2.401 to -0.064) | p = 0.039 |
| **Duty-hours per week (hours)** |  |  |
| Category 1 (< 60), n (%) | Reference | Reference |
| Category 2 (60–79), n (%) | 0.601 (0.164 to 1.037) | p = 0.007 |
| Category 3 (≥ 80), n (%) | -0.088 (-0.670 to 0.494) | p = 0.767 |
