# Supplementary material for: Impact of inpatient volume on residents’ In-training examination scores and burnout in Japanese community hospitals: a nationwide cross-sectional study
Source: BMC Med Educ. 2026 Jan 24;26:409. doi: 10.1186/s12909-026-08664-3 (PMC12980981; doi:10.1186/s12909-026-08664-3)
Supplement: Supplementary file 6 — Supplementary Material 6. [file 12909_2026_8664_MOESM6_ESM.docx]

**Supplemental 6:** Sensitivity analysis: Multilevel logistic regression for burnout symptoms including yearly inpatient volume and minimally adjusted hospital- and resident-level covariates.

| **Factors** | Adjusted odds ratio (95% CI) | p-value |
| --- | --- | --- |
| **Hospital-level information** |  |  |
| **Average number of inpatients** |  |  |
| Very Low-Volume Hospitals | Reference | Reference |
| Low-Volume Hospitals | 1.015 (0.514 to 2.005) | p = 0.965 |
| Moderate-Volume Hospitals | 1.038 (0.529 to 2.035) | p = 0.914 |
| High-Volume Hospitals | 1.123 (0.555 to 2.270) | p = 0.747 |
| **Number of permitted beds** | 0.994 (0.916 to 1.078) | p = 0.880 |
| **Annual number of CT scans** | 1.002 (0.991 to 1.014) | p = 0.673 |
| **Annual number of MRI scans** | 1.002 (0.977 to 1.027) | p = 0.878 |
| **Resident-level information** |  |  |
| **Grade** |  |  |
| PGY-1 | Reference | Reference |
| PGY-2 | 1.038 (0.873 to 1.235) | p = 0.671 |
| **Gender** |  |  |
| Men | Reference | Reference |
| Women | 1.134 (0.938 to 1.371) | p = 0.193 |
| **Average number of assigned inpatients** |  |  |
| 0-4 | Reference | Reference |
| 5-9 | 1.047 (0.862 to 1.270) | p = 0.645 |
| 10-14 | 0.811 (0.563 to 1.168) | p = 0.261 |
| ≥ 15 | 0.483 (0.293 to 0.796) | p = 0.004 |
| Unknown | 1.130 (0.612 to 2.085) | p = 0.697 |
| **Night shifts per month** |  |  |
| 0 | Reference | Reference |
| 1-2 | 1.054 (0.537 to 2.070) | p = 0.879 |
| 3-5 | 1.219 (0.642 to 2.314) | p = 0.545 |
| ≥ 6 | 1.265 (0.629 to 2.541) | p = 0.510 |
| Unknown | 0.462 (0.080 to 2.682) | p = 0.389 |
| **Self-study time per day (minutes)** |  |  |
| 1-30 | Reference | Reference |
| 31-60 | 1.131 (0.934 to 1.369) | p = 0.207 |
| 61-90 | 1.119 (0.839 to 1.490) | p = 0.444 |
| ≥ 91 | 1.306 (0.715 to 2.386) | p = 0.384 |
| 0 | 0.869 (0.530 to 1.427) | p = 0.579 |
| **Duty-hours per week (hours)** |  |  |
| Category 1 (< 60), n (%) | Reference | Reference |
| Category 2 (60–79), n (%) | 0.797 (0.652 to 0.975) | p = 0.027 |
| Category 3 (≥ 80), n (%) | 0.666 (0.517 to 0.857) | p = 0.002 |
