# Supplementary material for: Impact of inpatient volume on residents’ In-training examination scores and burnout in Japanese community hospitals: a nationwide cross-sectional study
Source: BMC Med Educ. 2026 Jan 24;26:409. doi: 10.1186/s12909-026-08664-3 (PMC12980981; doi:10.1186/s12909-026-08664-3)
Supplement: Supplementary file 7 — Supplementary Material 7. [file 12909_2026_8664_MOESM7_ESM.docx]

**Supplemental 7:** Sensitivity analysis: Multilevel logistic regression for burnout symptoms adjusted for yearly inpatient volume and resident-level covariates only.

| **Factors** | Adjusted odds ratio (95% CI) | p-value |
| --- | --- | --- |
| **Hospital-level information** |  |  |
| **Average number of inpatients** |  |  |
| Very Low-Volume Hospitals | Reference | Reference |
| Low-Volume Hospitals | 1.017 (0.516 to 2.006) | p = 0.960 |
| Moderate-Volume Hospitals | 1.051 (0.540 to 2.046) | p = 0.884 |
| High-Volume Hospitals | 1.160 (0.602 to 2.235) | p = 0.657 |
| **Resident-level information** |  |  |
| **Grade** |  |  |
| PGY-1 | Reference | Reference |
| PGY-2 | 1.038 (0.872 to 1.234) | p = 0.675 |
| **Gender** |  |  |
| Men | Reference | Reference |
| Women | 1.134 (0.938 to 1.370) | p = 0.195 |
| **Average number of assigned inpatients** |  |  |
| 0-4 | Reference | Reference |
| 5-9 | 1.046 (0.862 to 1.269) | p = 0.648 |
| 10-14 | 0.812 (0.564 to 1.169) | p = 0.263 |
| ≥ 15 | 0.488 (0.298 to 0.799) | p = 0.004 |
| Unknown | 1.126 (0.610 to 2.076) | p = 0.705 |
| **Night shifts per month** |  |  |
| 0 | Reference | Reference |
| 1-2 | 1.053 (0.538 to 2.063) | p = 0.880 |
| 3-5 | 1.218 (0.642 to 2.309) | p = 0.547 |
| ≥ 6 | 1.275 (0.636 to 2.558) | p = 0.494 |
| Unknown | 0.465 (0.080 to 2.692) | p = 0.393 |
| **Self-study time per day (minutes)** |  |  |
| 1-30 | Reference | Reference |
| 31-60 | 1.130 (0.933 to 1.368) | p = 0.211 |
| 61-90 | 1.115 (0.837 to 1.485) | p = 0.456 |
| ≥ 91 | 1.304 (0.714 to 2.379) | p = 0.388 |
| 0 | 0.867 (0.529 to 1.423) | p = 0.573 |
| **Duty-hours per week (hours)** |  |  |
| Category 1 (< 60), n (%) | Reference |  |
| Category 2 (60–79), n (%) | 0.798 (0.652 to 0.975) | p = 0.027 |
| Category 3 (≥ 80), n (%) | 0.669 (0.520 to 0.860) | p = 0.002 |
