# Supplementary material for: Impact of inpatient volume on residents’ In-training examination scores and burnout in Japanese community hospitals: a nationwide cross-sectional study
Source: BMC Med Educ. 2026 Jan 24;26:409. doi: 10.1186/s12909-026-08664-3 (PMC12980981; doi:10.1186/s12909-026-08664-3)
Supplement: Supplementary file 8 — Supplementary Material 8. [file 12909_2026_8664_MOESM8_ESM.docx]

**Supplemental 8:** Sensitivity analysis: Multilevel linear regression for GM-ITE^®^ scores treating yearly inpatient volume as a continuous variable.

| **Factors** | Adjusted estimated coefficient (95% CI) | p-value |
| --- | --- | --- |
| **Hospital-level information** |  |  |
| **Average number of inpatients** | 0.003 (-0.002 to 0.008) | p = 0.297 |
| **Number of permitted beds** | -0.252 (-0.627 to 0.123) | p = 0.187 |
| **Annual number of ambulances** | 0.011 (-0.036 to 0.059) | p = 0.641 |
| **Annual number of outpatients** | 0.050 (-0.056 to 0.156) | p = 0.359 |
| **Number of days in hospital** | -0.013 (-0.057 to 0.031) | p = 0.556 |
| **Number of doctors** | 0.619 (-0.237 to 1.476) | p = 0.156 |
| **Number of nurses** | 0.294 (-0.031 to 0.620) | p = 0.077 |
| **Annual number of CT scans** | -0.056 (-0.094 to -0.018) | p = 0.004 |
| **Annual number of MRI scans** | 0.037 (-0.025 to 0.099) | p = 0.239 |
| **Resident-level information** |  |  |
| **Grade** |  |  |
| PGY-1 | Reference | Reference |
| PGY-2 | 1.675 (1.297 to 2.053) | p < 0.001 |
| **Gender** |  |  |
| Men | Reference | Reference |
| Women | 0.009 (-0.394 to 0.413) | p = 0.964 |
| **Average number of assigned inpatients** |  |  |
| 0-4 | Reference | Reference |
| 5-9 | 1.041 (0.610 to 1.471) | p < 0.001 |
| 10-14 | 0.829 (-0.052 to 1.710) | p = 0.065 |
| ≥ 15 | 0.474 (-0.993 to 1.941) | p = 0.527 |
| Unknown | 0.386 (-0.917 to 1.689) | p = 0.562 |
| **Night shifts per month** |  |  |
| 0 | Reference | Reference |
| 1-2 | 0.465 (-1.095 to 2.025) | p = 0.559 |
| 3-5 | 0.599 (-0.885 to 2.084) | p = 0.429 |
| ≥ 6 | 0.440 (-1.192 to 2.072) | p = 0.597 |
| Unknown | 3.022 (-1.781 to 7.824) | p = 0.217 |
| **Self-study time per day (minutes)** |  |  |
| 1-30 | Reference | Reference |
| 31-60 | 0.683 (0.270 to 1.097) | p = 0.001 |
| 61-90 | 1.191 (0.565 to 1.818) | p < 0.001 |
| ≥ 91 | 1.339 (0.091 to 2.586) | p = 0.036 |
| 0 | -1.244 (-2.413 to -0.074) | p = 0.037 |
| **Duty-hours per week (hours)** |  |  |
| Category 1 (< 60), n (%) | Reference | Reference |
| Category 2 (60–79), n (%) | 0.594 (0.157 to 1.030) | p = 0.008 |
| Category 3 (≥ 80), n (%) | -0.122 (-0.705 to 0.461) | p = 0.682 |
