# Supplementary material for: Impact of inpatient volume on residents’ In-training examination scores and burnout in Japanese community hospitals: a nationwide cross-sectional study
Source: BMC Med Educ. 2026 Jan 24;26:409. doi: 10.1186/s12909-026-08664-3 (PMC12980981; doi:10.1186/s12909-026-08664-3)
Supplement: Supplementary file 9 — Supplementary Material 9. [file 12909_2026_8664_MOESM9_ESM.docx]

**Supplemental 9:** Sensitivity analysis: Multilevel logistic regression for burnout symptoms treating yearly inpatient volume as a continuous variable.

| **Factors** | Adjusted odds ratio (95% CI) | p-value |
| --- | --- | --- |
| **Hospital-level information** |  |  |
| **Average number of inpatients** | 0.999 (0.997 to 1.001) | p = 0.256 |
| **Number of permitted beds** | 1.007 (0.877 to 1.156) | p = 0.922 |
| **Annual number of ambulances** | 1.025 (1.007 to 1.043) | p = 0.006 |
| **Annual number of outpatients** | 1.029 (0.991 to 1.068) | p = 0.132 |
| **Number of days in hospital** | 1.001 (0.982 to 1.021) | p = 0.891 |
| **Number of doctors** | 1.186 (0.877 to 1.604) | p = 0.268 |
| **Number of nurses** | 0.929 (0.822 to 1.048) | p = 0.231 |
| **Annual number of CT scans** | 0.994 (0.980 to 1.007) | p = 0.348 |
| **Annual number of MRI scans** | 1.003 (0.978 to 1.028) | p = 0.810 |
| **Resident-level information** |  |  |
| **Grade** |  |  |
| PGY-1 | Reference | Reference |
| PGY-2 | 1.042 (0.876 to 1.240) | p = 0.641 |
| **Gender** |  |  |
| Men | Reference | Reference |
| Women | 1.130 (0.935 to 1.366) | p = 0.207 |
| **Average number of assigned inpatients** |  |  |
| 0-4 | Reference | Reference |
| 5-9 | 1.049 (0.864 to 1.274) | p = 0.631 |
| 10-14 | 0.797 (0.553 to 1.148) | p = 0.223 |
| ≥ 15 | 0.447 (0.270 to 0.742) | p = 0.002 |
| Unknown | 1.125 (0.609 to 2.078) | p = 0.707 |
| **Night shifts per month** |  |  |
| 0 | Reference | Reference |
| 1-2 | 1.072 (0.546 to 2.104) | p = 0.840 |
| 3-5 | 1.236 (0.652 to 2.344) | p = 0.517 |
| ≥ 6 | 1.247 (0.621 to 2.503) | p = 0.535 |
| Unknown | 0.493 (0.085 to 2.872) | p = 0.431 |
| **Self-study time per day (minutes)** |  |  |
| 1-30 | Reference | Reference |
| 31-60 | 1.130 (0.933 to 1.368) | p = 0.211 |
| 61-90 | 1.130 (0.848 to 1.507) | p = 0.404 |
| ≥ 91 | 1.300 (0.711 to 2.375) | p = 0.394 |
| 0 | 0.879 (0.535 to 1.445) | p = 0.611 |
| **Duty-hours per week (hours)** |  |  |
| Category 1 (< 60), n (%) | Reference | Reference |
| Category 2 (60–79), n (%) | 0.787 (0.644 to 0.963) | p = 0.020 |
| Category 3 (≥ 80), n (%) | 0.646 (0.501 to 0.832) | p < 0.001 |
