# Supplementary material for: Impact of inpatient volume on residents’ In-training examination scores and burnout in Japanese community hospitals: a nationwide cross-sectional study
Source: BMC Med Educ. 2026 Jan 24;26:409. doi: 10.1186/s12909-026-08664-3 (PMC12980981; doi:10.1186/s12909-026-08664-3)
Supplement: Supplementary file 10 — Supplementary Material 10. [file 12909_2026_8664_MOESM10_ESM.docx]

**Supplemental 10:** The relationship between yearly inpatient volume and resident training guidance system.

|  | High (%) | Moderate (%) | Low (%) | Very Low (%) |
| --- | --- | --- | --- | --- |
| Strongly disagree | 0.75 | 1.27 | 0.97 | 3.37 |
| Disagree | 3.89 | 5.81 | 6.82 | 4.49 |
| Neither agree nor disagree | 12.67 | 14.88 | 12.8 | 14.61 |
| Agree | 53.28 | 50.76 | 50.21 | 59.55 |
| Strongly agree | 29.41 | 27.29 | 29.21 | 17.98 |
