# Supplementary material for: Impact of inpatient volume on residents’ In-training examination scores and burnout in Japanese community hospitals: a nationwide cross-sectional study
Source: BMC Med Educ. 2026 Jan 24;26:409. doi: 10.1186/s12909-026-08664-3 (PMC12980981; doi:10.1186/s12909-026-08664-3)
Supplement: Supplementary file 11 — Supplementary Material 11. [file 12909_2026_8664_MOESM11_ESM.docx]

**Supplemental 11:** The relationship between yearly inpatient volume and clinical training support system.

|  | High (%) | Moderate (%) | Low (%) | Very Low (%) |
| --- | --- | --- | --- | --- |
| Strongly disagree | 0.93 | 1.43 | 1.53 | 6.74 |
| Disagree | 3.68 | 5.89 | 7.51 | 6.74 |
| Neither agree nor disagree | 12.24 | 15.51 | 14.46 | 17.98 |
| Agree | 51.07 | 49.4 | 47.01 | 52.81 |
| Strongly agree | 32.08 | 27.76 | 29.49 | 15.73 |
