# Supplementary material for: Impact of inpatient volume on residents’ In-training examination scores and burnout in Japanese community hospitals: a nationwide cross-sectional study
Source: BMC Med Educ. 2026 Jan 24;26:409. doi: 10.1186/s12909-026-08664-3 (PMC12980981; doi:10.1186/s12909-026-08664-3)
Supplement: Supplementary file 12 — Supplementary Material 12. [file 12909_2026_8664_MOESM12_ESM.docx]

**Supplemental 12:** The relationship between yearly inpatient volume and academic journal availability.

|  | High (%) | Moderate (%) | Low (%) | Very Low (%) |
| --- | --- | --- | --- | --- |
| Strongly disagree | 1.89 | 3.1 | 2.23 | 2.25 |
| Disagree | 7.28 | 9.23 | 10.71 | 15.73 |
| Neither agree nor disagree | 18.2 | 23.07 | 24.06 | 21.35 |
| Agree | 46.25 | 43.2 | 42.7 | 44.94 |
| Strongly agree | 26.37 | 21.4 | 20.31 | 15.73 |
